# An easy assembled fluorescent sensor for dicarboxylates and acidic amino acids

**DOI:** 10.3762/bjoc.7.11

**Published:** 2011-01-17

**Authors:** Xiao-bo Zhou, Yuk-Wang Yip, Wing-Hong Chan, Albert W M Lee

**Affiliations:** 1 Department of Chemistry, Hong Kong Baptist University, Kowloon Tong, Hong Kong SAR, China

**Keywords:** amino acids, dicarboxylate ion recognition, enantioselectivity, mesitylene scaffold

## Abstract

Two mesitylene based neutral receptors **1** and **2** bearing two thiourea binding sites were constructed as fluorescent probes for sensing dicarboxylates. Their binding affinities toward dicarboxylates, aspartate and glutamate have been investigated in acetonitrile solution by fluorescence titration experiments. Both fluorescent sensors exhibited some ability to discriminate the antipodal forms of aspartate and glutamate.

## Introduction

The recognition and sensing of anionic substrates by positively charged or electrically neutral synthetic receptive molecular systems has emerged as a key research area because of the fundamental roles of anions in many chemical and biological processes [1–5]. Being the key structural moieties of many bioactive molecules such as amino acids and proteins, dicarboxylates are one of the most attractive targets for anion recognition and sensing. In addition, dicarboxylates are essential components of a variety of metabolic processes such as the generation of high-energy phosphate bonds and the biosynthesis of important intermediates [6–7]. In recent years, numerous endeavors have been devoted to the design and synthesis of ditopic anion receptors bearing optical signal display subunits (i.e., chromophore or fluorophore) as sensing probes for dicarboxylates [8–13]. Additionally, chiral recognition of carboxylates has been actively explored in the sensor field [14–16]. By using cholic acid as the molecular scaffold for the construction of sensing probes, we have developed fluorescent probes for detecting dicarboxylates and trifunctional aminoacids [17–18]. To continue our interest in this research direction, we report here the facile synthesis and molecular recognition properties of two new sensing probes **1** and **2**.

Trimethyl- or triethylbenzene have been widely used building blocks to prepare both tripodal or ditopic supramolecular systems for molecular recognition [19–24]. An obvious incentive for the use of the mesitylyl moiety is that the required 1,3,5-trimethyl-2,4-bis(bromomethyl)benzene and 1,3,5-trimethyl-2,4,6-tri(bromomethyl)benzene as starting materials are readily prepared [25]. Upon suitable functionalization, the three alkyl groups of the substituted mesitylene would preorganize towards the same face of the benzene core to favor the formation of a semi-rigid conformation [26]. We thus chose to employ this mesitylene derivative as our starting material for the design and synthesis of dicarboxylate sensors.

## Results and Discussion

**Scheme 1:**
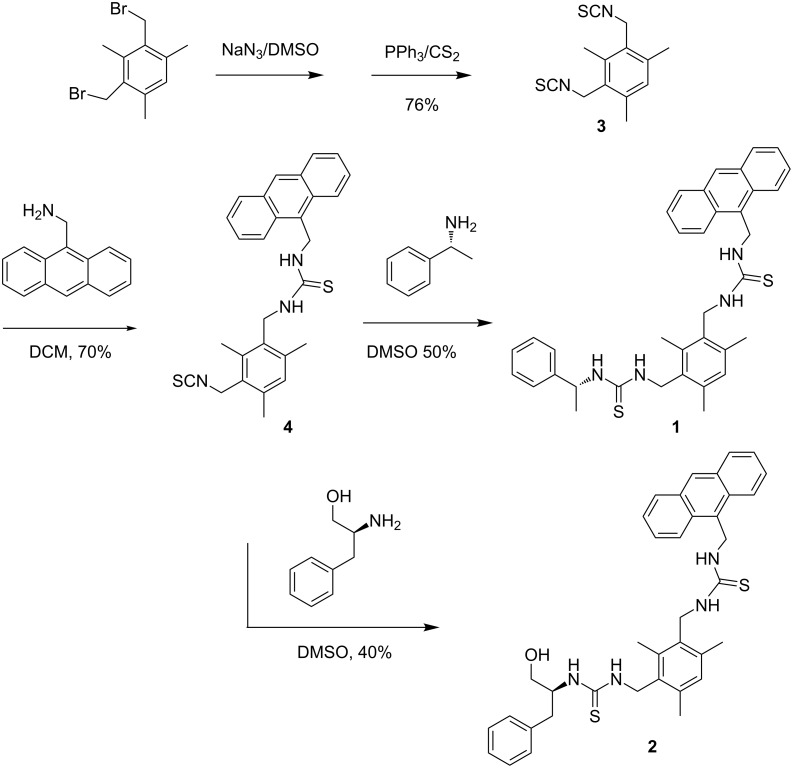
Synthesis of sensor **1** and **2**.

The 2-step protocol developed by Banert et al. [27] was adopted for the synthesis of the sensors. Thus nucleophilic substitution of the bromine atoms of 1,3-bis[bromomethyl]-2,4,6-trimethylbenzene with sodium azide in DMSO afforded the corresponding bis-azide. Transformation of the crude bis-azide into bis-isothiocyanate **3** was achieved by treatment with triphenylphosphine in the presence of CS_2_. The “fluorophore–spacer–receptor” sensing motif for carboxylate was incorporated into the molecular platform via thiourea formation to give the key intermediate **4** in 70% yield. As a result of steric hindrance, an attempt to make the corresponding bis-thiourea adduct from **3** and 9-aminomethylanthracene was unsuccessful. However, **4** underwent a smooth addition reaction with the structurally less bulky (+)-α-ethylphenylamine to afford sensor **1** in 50% yield (Scheme 1). It is noteworthy that by appending a chiral element onto the sensor scaffold, **1** may demonstrate enantioselectivity towards optically active materials such as amino acids. In continuation of the pioneering work of Gunnlaugsson, the charge neutral thiourea-based fluorescent PET sensor motif, which is immune from cross pH interference, was adopted in our approach [28]. The congested mesitylene derivative type of structure built in **1** may confer the molecule with sufficient rigidity favoring its selective binding with guest molecules. The receptor **1** was fully characterized by ^1^H, ^13^C NMR, and high resolution mass spectral analysis.

**Figure 1:**
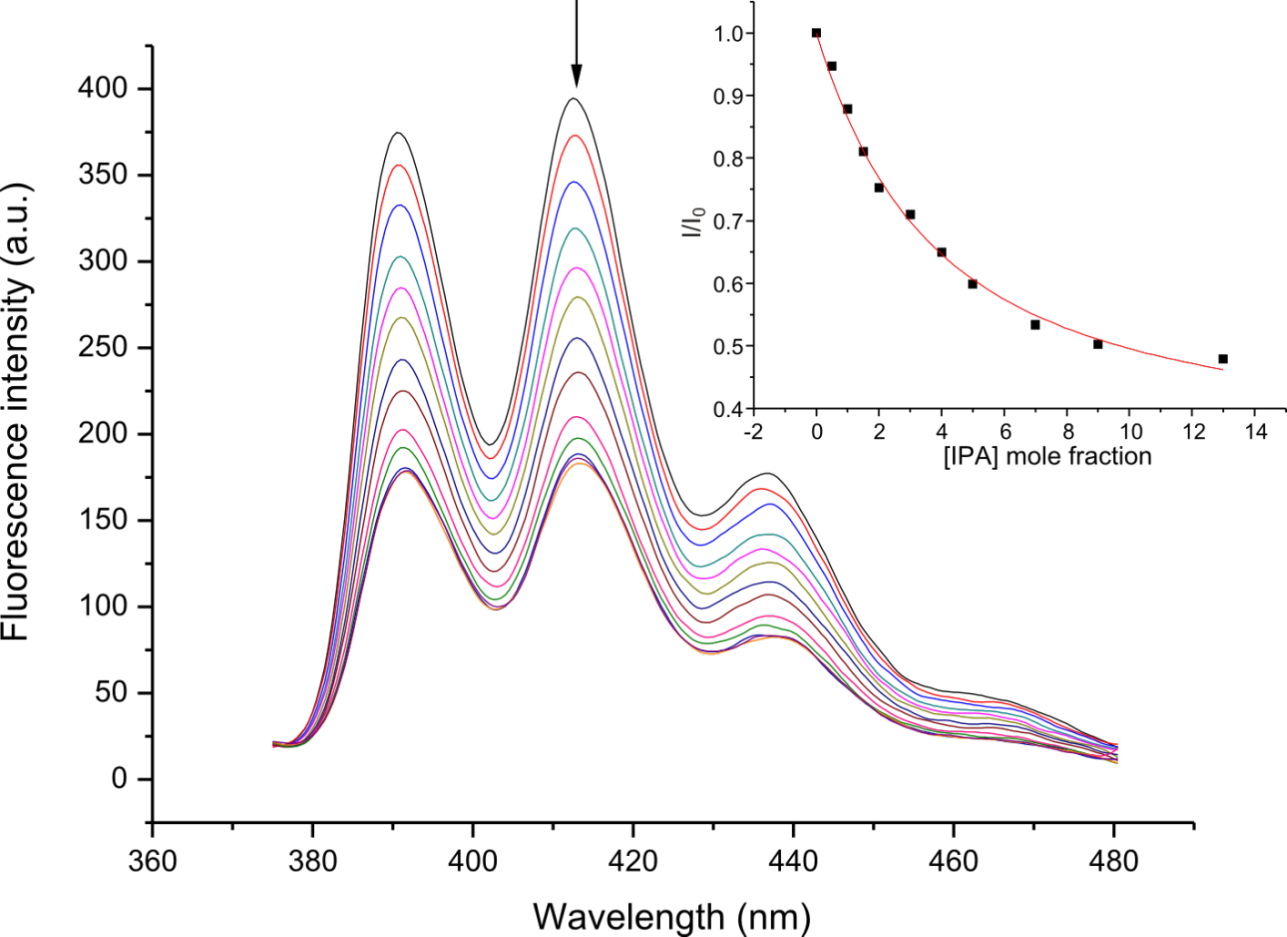
The changes in the fluorescence emission spectra of sensor **1** (5.0 × 10^−6^ M) upon addition of isophthalate in acetonitrile. λ_ex_ = 366 nm. (Inset) Quenching ratio of sensor **1** as a function of (guest)/(host).

To evaluate the binding affinity of the synthetic host to dicarboxylate guest molecules, fluorometric titration experiments were carried out with the concentration of **1** fixed at 5.0 × 10^−6^ M in acetonitrile and the guest concentration (as tetrabutylammonium salts) was varied from 5.0 × 10^−6^ M to 1.0 × 10^−4^ M. The typical change of emission spectra of **1** caused by isophthalate is shown in Figure 1. Operating on the PET mechanism, the fluorescent emission band of the host at 413 nm was quenched gradually upon the addition of the guest.

On the basis of a Job plot, a 1:1 complex between **1** and isophthalate was confirmed (Figure S1, Supporting Information File 1). To give a full picture of the binding characteristics of the synthetic host, other aromatic and aliphatic dicarboxylates were included in the study. By analyzing the change of fluorescent intensity associated with the stepwise addition of guest dicarboxylates, the “host–guest” complex association constants (*K*_a_) were calculated using non-linear least-squares curve fitting and the results are compiled in Table 1. It is noteworthy that acetate being a monoanion did not cause any fluorescence quenching to sensor **1** (Figure S2, Supporting Information File 1).

**Table 1:** Association constants *K*_ass_ (M^−1^) of sensor **1** with various dicarboxylates (as their tetrabutylammonium salts) in CH_3_CN.

Anion	*K*_ass_^a^	R^2^

Isophthalate	(6.25 ± 0.61) × 10^4^	0.9949
Phthalate	(1.34 ± 0.11) × 10^4^	0.9955
Terephthalate	(1.23 ± 0.58) × 10^4^	0.9835
Oxalate	(2.16 ± 0.12) × 10^4^	0.9974
Malonate	(2.49 ± 0.24) × 10^4^	0.9927
Succinate	(1.95 ± 0.21) × 10^4^	0.9909
Glutarate	(1.60 ± 0.24) × 10^4^	0.9857

^a^*K*_ass_ is the apparent constant for the equilibrium of the 1:1 stoichiometric ratio between **1** and dicarboxylate in CH_3_CN.

On the basis of the fairly similar association constants between **1** and different dicarboxylates, the two tweezer-like thiourea side-arms of **1** seem to be quite flexible and are able to accommodate dicarboxylates of different chain lengths. The 3–4 fold stronger binding interaction between sensor **1** and isophthalate in comparison with those of other dicarboxylates may be the result of the good host–guest shape-complementary relationship and the π–π interactions between the aromatic moieties of the host and the guest. To examine the chiral discrimination ability of sensor **1** toward chiral acidic aminoacids, fluorescence titrations of sensor **1** with antipodal aspartates and glutamates were carried out separately. Using succinate as the reference guest molecule, the additional amino group present in aspartates weakened their interaction with sensor **1**, presumably due to non-bonding repulsions.

**Figure 2:**
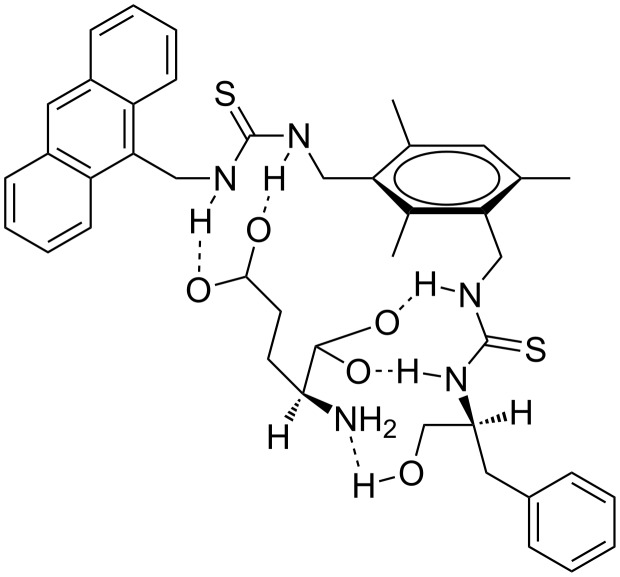
Proposed three sites binding model for sensor **2** and glutamate complex.

In contrast, in comparison with the association constant between sensor **1** and glutarate, sensor **1** exhibited a stronger binding affinity to D- and L-glutamate (Table 2). Interestingly, a marginal enantioselectivity toward the antipodal forms of aminoacids demonstrated by sensor **1** is evident. We were aware that sensor **1** can only exhibit two-point interaction with aspartate and glutamate via the thiourea–carboxylate type of binding motif. For the host to exert a greater influence on the preorganization of the guests, an additional binding site must be introduced. Thus, (*S*)-phenylalaninol (obtainable from L-phenylalanine) was reacted with isothiocyanate **4** to give sensor **2** in 40% yield. We envisaged that the additional alcohol functionality present in **2** could provide a binding site for the α-amino group of aspartate and glutamate. Experimental findings indeed corroborated well with such a supposition. Compared with the interaction of the control compounds (i.e., succinate and glutarate), sensor **2** demonstrated a 3–10 fold stronger binding affinity with aspartate and glutamate, respectively. For instance, the association constant of sensor **2** and D-glutamate is one order of magnitude greater than that of sensor **2** and glutarate. In addition to the carboxylate–thiourea interactions, additional H-bond interactions could arise from the alcohol group of the host and the amino group of the guest. A three site binding model is proposed (Figure 2) to rationalize the enhanced binding interaction between sensor **2** and glutamate.

**Table 2:** Association constants *K*_ass_ (M^−1^) of sensor **1** and **2** with antipodal aspartate and glutamate (as their tetrabutylammonium salts) in CH_3_CN.

	Sensor **1**		Sensor **2**	

Anion	*K*_ass_	Enantioselectivity	*K*_ass_	Enantioselectivity

Succinate	(1.95 ± 0.21) × 10^4^	—	(0.34 ± 0.03) × 10^4^	—
D-Aspartate	(0.57 ± 0.05) × 10^4^		(2.13 ± 0.31) × 10^4^	
L-Aspartate	(1.33 ± 0.15) × 10^4^	*K*_L_/*K*_D_ = 2.3	(1.19 ± 0.25) × 10^4^	*K*_L_/*K*_D_ = 0.56
Glutarate	(1.60 ± 0.24) × 10^4^	—	(0.52 ± 0.05) × 10^4^	—
D-Glutamate	(2.05 ± 0.05) × 10^4^		(5.17 ± 0.31) × 10^4^	
L-Glutamate	(3.73 ± 0.08) × 10^4^	*K*_L_/*K*_D_ = 1.8	(3.35 ± 0.36) × 10^4^	*K*_L_/*K*_D_ = 0.65

In comparison with sensor **1**, sensor **2** exhibited reversed enantiomeric bias towards aspartate and glutamate. On the other hand, it was quite surprising to see that the enantioselectivity demonstrated by sensor **2** toward the antipodal forms of the aminoacids was very small. Apparently, the rigidity of the host molecule is not sufficient to generate a unique binding cavity so as to effectively differentiate between the two enantiomeric acidic aminoacids.

**Figure 3:**
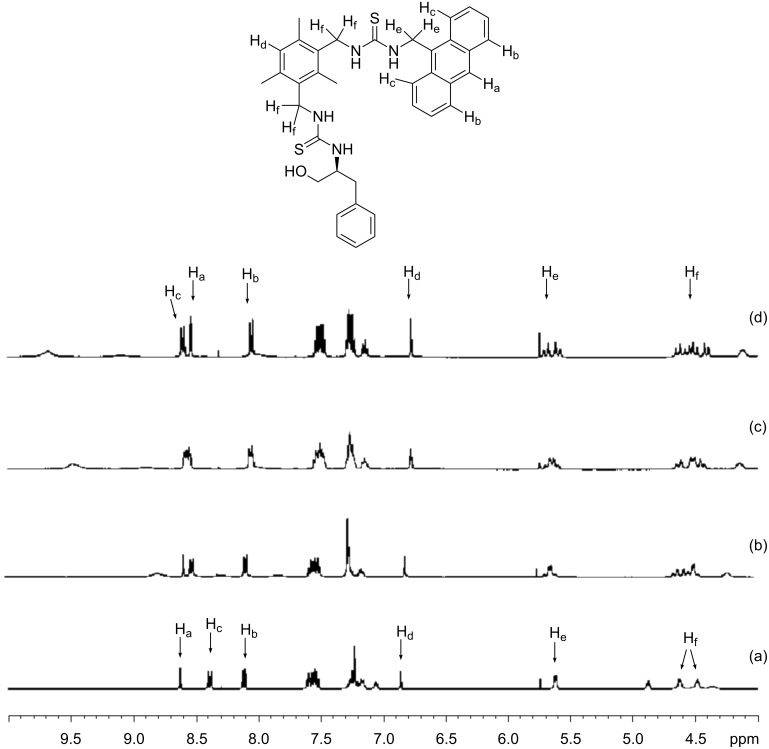
Partial ^1^H NMR spectra for (a) sensor **2** (free, 3 mM), (b) sensor **2** + 0.5 equiv D-glutamate, (c) sensor **2** + 1.0 equiv D-glutamate, (d) sensor **2** + 1.0 equiv L-glutamate in DMSO-*d*_6_.

To shed some light on the binding interaction between sensor **2** and glutamate, an ^1^H NMR spectroscopic method was employed. By adding one equivalent of either D- or L-glutamate to a DMSO solution of sensor **2**, as shown in Figure 3, the resultant ^1^H NMR spectra displayed two distinct changes. The broad singlets at δ 5.62, 4.69 and 4.49 ascribed to anthracenyl and the two benzylic methylene protons, respectively, are split into three sets of doublet of doublets. Apparently, after coordinating with the guest, bond rotation of host **2** was restricted, with the result that the two methylene protons adjacent to the aromatic ring (i.e., H_e_ and H_f_) became magnetically non-equivalent. At the same time, the H_a_, H_b_ and H_d_ protons of the host experienced an upfield shift of ~0.07 ppm, whereas the H_c_ protons were downfield shifted by 0.19 ppm. Conceivably, as a result of complexation, the spatial relationship between the mesitylene and the anthracene moiety of the host may slightly change, inducing a change in the chemical shifts for some protons. On the other hand, the guest induced shift of selected protons of sensor **2** caused by D- and L-glutamate was almost identical, which is consistent with the low enantioselectivity exhibited by the host. Interestingly, when 0.5 equiv of D-glutamate was added to the host, one of the benzylic methylene protons of the host became a broad singlet. This could be rationalized as follows: The thiourea group adjacent to the phenylalaninol moiety possessing two binding sites first complexes with the α-aminocarboxylate group of glutamate. On binding, the mobility of the corresponding pendant side-arm is reduced thus leading to the broad singlet centered at δ 4.69 splitting into a multiplet (Figure 3b). The observation corroborates well with our binding model shown in Figure 2.

To address the issue of selectivity of sensor **1** towards other anions, fluorescence titrations of **1** were conducted with dihydrogen phosphate, nitrate and bromide. Upon addition of up to 20 equiv of these anions separately to the sensor solutions, no fluorescence change of sensor **1** was observed (Figure S3, Supporting Information Information File 1). On the other hand, among common transition metal ions (i.e., Ag^+^, Zn^2+^, Cd^2+^, Fe^2+^, Co^2+^, Ni^2+^, Hg^2+^, Pb^2+^, Cu^2+^), only Cu(II) led to suppression of the fluorescence of sensor **1** (Figure S4, Supporting Information File 1). Apparently, sensor **1** is a fairly selective probe for detecting dicarboxylates.

## Conclusion

This study examined the binding properties of two ditopic receptors **1** and **2** for various dicarboxylates using fluorescence and ^1^H NMR spectroscopic methods. As revealed by the respective association constants, among all studied dicarboxylates, sensor **1** showed the highest affinity to isophthalate. The preorganization of sensor **2** permits three site binding with the guests leading to enhanced complexation with aspartate and glutamate in contrast to their corresponding dicarboxylate counterparts. The chiral recognition ability of the sensors is however, only moderate. In order to be a better enantioselective dicarboxylate sensor, the flexibility of the two thiourea receptive sites of the host must be constrained. Design of new enantioselective sensors along this direction is underway.

## Experimental

**General.** Melting points were determined with a MEL-TEMPII melting point apparatus and are uncorrected. ^1^H and ^13^C NMR spectra were recorded on a Bruker Avance-III spectrometer (at 400 and 100 MHz, respectively) in DMSO-*d*_6_ or CDCl_3_. High resolution mass spectra were recorded on a Bruker Autoﬂex mass spectrometer (MALDI TOF). Fluorescent emission spectra were taken on a Perkin Elmer LS 50B luminescence spectroﬂuorimeter. Unless otherwise specified, all fine chemicals were used as received.

**Synthesis of 2,4-bis(isothiocyanatomethyl)-1,3,5-trimethylbenzene (3):** A mixture of 3,4-bis(bromomethyl)-1,3,5-trimethylbenzene (0.306 g, 1 mmol) and NaN_3_ (0.14 g, 2.2 mmol) in dry DMSO (30 mL) was stirred for 3 h at 50 °C. After cooling to room temperature, PPh_3_ (0.58 g, 2.2 mmol) was added with cooling. The mixture was stirred for 16 h at room temperature and CS_2_ (0.18 mL, 3.0 mmol) added dropwise to the ice-cooled reaction mixture. When the vigorous reaction had subsided, the mixture was stirred for an additional 4 h at room temperature, then poured into water, and extracted repeatedly with DCM (3 × 30 mL). The combined organic layers were washed several times with water, dried with MgSO_4_ and filtered. After removal of the solvent in vacuo, the residue was purified by column chromatography on SiO_2_ with chloroform:PE (1:10) as eluent to give **3** as a white solid (200 mg, 76% yield). Its characterization data is in agreement with that reported [29].

**Synthesis of 2-anthrancenylthioureamethyl-4-isothiocyanatomethyl-1,3,5-trimethylbenzene (4):** Compound **3** (130 mg, 0.5 mmol) and anthrancen-9-yl-methanamine (110 mg, 0.5 mmol) were dissolved in freshly distilled dry dichloromethane (10 mL) and the mixture was stirred for 14 h. The crude product collected after work-up was purified by column chromatography on SiO_2_ with chloroform:PE (2:1) as eluent to give **4** as a white solid (60 mg, 70% yield), mp 129–133 °C.

^1^H NMR (400 MHz, CDCl_3_): δ 2.26 (s, 3H), 2.27 (s, 3H), 2.30 (s, 3H), 4.64 (s, 2H), 4.82 (s, 2H), 5.64 (s, 2H), 6.93 (s, 1H), 7.16 (br, 1H), 7.55-7.63 (m, 4H), 8.12 (d, 2H, *J* = 8.4 Hz), 8.41(d, 2H, *J* = 8.8Hz), 8.63 (s, 1H). ^13^C NMR (100 MHz, CDCl_3_): δ 15.19, 19.11, 19.56, 40.63, 43.24, 124.29, 125.29, 126.50, 127.51, 128.27, 128.92, 129.39, 129.45, 129.87, 130.03, 130.97, 133.00, 136.19, 136.61, 137.68, 181.84. MALDI TOF HRMS: calcd for C_28_H_27_N_3_S_2_ [M+H]^+^ 470.1719; found 470.1700.

**Synthesis of Sensor 1:** Compound **4** (70 mg, 0.15 mmol) and (+)-α-ethylphenylamine (24 mg, 0.2 mmol) were dissolved in dry DMSO (10 mL) and the mixture was stirred for 14 h at 100 °C. The crude product collected after work-up was purified by column chromatography on SiO_2_ with chloroform as eluent to give **1** as a yellow solid (45 mg, 50% yield), mp 244–246 °C. [α]^25^_D_ = −30.1 (c = 0.65, DMSO).

^1^H NMR (400 MHz, CDCl_3_): δ 1.35 (d, 2H, *J* = 6.8 Hz), δ 2.21(s, 6H), 2.26 (s, 3H), 4.64 (s, 2H), 4.82 (s, 2H), 5.64 (s, 2H), 6.93 (s, 1H), 7.16 (br, 1H), 7.55-7.63 (m, 4H), 8.12 (d, 2H, *J* = 8.4 Hz), 8.41(d, 2H, *J* = 8.4 Hz), 8.63 (s, 1H). ^13^C NMR (100 MHz, CDCl_3_): δ 15.19, 19.11, 19.56, 40.63, 43.24, 124.29, 125.29, 126.50, 127.51, 128.27, 128.92, 129.39, 129.45, 129.87, 130.03, 130.97, 133.00, 136.19, 136.61, 137.68, 181.84. MALDI TOF HRMS: calcd for C_36_H_38_N_4_S_2_ [M+H]^+^ 591.2583; found 591.2596.

**Synthesis of sensor 2:** Compound **4** (70 mg, 0.15mmol ) and (*S*)-phenylalaninol (30 mg, 0.2 mmol) were dissolved in dry DMSO (10 mL) and the mixture was stirred for 14 h at 100 °C. The crude product collected after work-up was purified by column chromatography on SiO_2_ with chloroform as eluent to give **2** as a yellow solid (36 mg, 40% yield): mp 224–225 °C. [α]^21^_D_ = −16.3 (c = 0.50, DMSO).

^1^H NMR (400 MHz, CDCl_3_): δ 2.21(s, 6H), 2.24 (s, 3H), 2.76–2.78(m, 2H), 3.30–3.33(m, 2H), 4.36(s, 1H), 4.48(s, 2H), 4.62 (s,2H), 4.87(t, 1H, *J* = 4.8 Hz), 5.62 (s, 2H), 6.86 (s, 1H), 7.05 (br, 1H), 7.06–7.27(m, 7H), 7.51–7.63 (m, 4H), 8.11 (d, 2H, J = 8.4 Hz), 8.39 (d, 2H, *J* = 8.4 Hz), 8.63 (s, 1H). ^13^C NMR (100 MHz,CDCl_3_): δ 15.39, 19.45, 19.51, 28.94, 36.36, 42.73, 56.37, 60.91, 124.27, 125.30, 125.95, 126.52, 127.52, 128.10, 128.93, 129.09, 129.44, 129.68, 129.86, 130.98, 132.55, 132.60, 136.24, 136.38, 136.77, 138.86, 181.82. MALDI TOF HRMS: calcd for C_37_H_40_N_4_OS_2_ [M+H]^+^ 621.2727; found 621.2732.

**Preparation of fluorometric anion titration solutions:** Stock solutions (5 mM) of the tetrabutylammonium salts of phthalatic acid, isophthalic acid, terephthalic acid, oxalic acid, malonic acid, succinic acid, glutaric acid, D- and L-aspartic acid, and D- and L-glutamic acid in CH_3_CN were prepared. Stock solutions of hosts (1 mM) were prepared in DMSO. Test solutions were prepared by adding 25 µL of the stock host solution and different volumes (5–100 µL) of the anion solution to a series of test tubes followed by dilution to 5 mL with acetonitrile. After being shaken for several minutes, the test solutions were measured immediately. For all measurements, the solutions were excited at 366 nm and emission was measured from 380–480 nm.

Association constants (1:1) of **1** and **2** with anions were calculated by non-linear least square curve ﬁtting using the following equation in Origin 7.5:


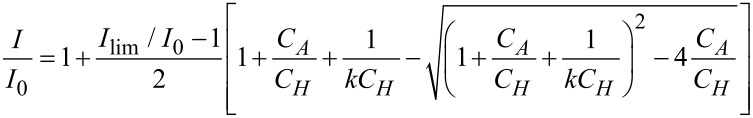


where *I*_0_ is fluorescent intensity of host without any anion, *I*_lim_ is fluorescent intensity limit on adding excess anion, *C_A_* is the concentration of the anion added, and *C_H_* is the concentration of the host molecule.

## Supporting Information

A Job plot of sensor **1** with isophthalate, interference studies and the ^1^H, ^13^C NMR and HRMS spectra of compound **1**, **2** and **4** are available as Supporting Information.

File 1 Spectral data of compounds **1**, **2** and **4** and Job plot of sensor **1**.
