# Supplementary material for: An easy assembled fluorescent sensor for dicarboxylates and acidic amino acids
Source: Beilstein J Org Chem. 2011 Jan 17;7:75–81. doi: 10.3762/bjoc.7.11 (PMC3028595; doi:10.3762/bjoc.7.11)
Supplement: File 1 — Spectral data of compounds 1, 2 and 4 and Job plot of sensor 1. [file Beilstein_J_Org_Chem-07-75-s001.pdf]

Supporting Information

for

An easy assembled fluorescent sensor for dicarboxylates and acidic amino acids

Xiao-bo Zhou, Yuk-Wang Yip, Wing-Hong Chan* and Albert W. M. Lee

Address: Department of Chemistry, Hong Kong Baptist University, Kowloon Tong, Hong Kong SAR, China

Email: Wing-Hong Chan - whchan@hkbu.edu.hk

*Corresponding author

Spectral data of compounds 1, 2 and 4 and Job plot of sensor 1

1. Figure S1, Figure S2 .... S2
2. Figure S3 .... S3
3. Figure S4 .... S4
4. ^{1}H NMR of compound **4** S5
5. ^{13}C NMR of compound **4** S6
6. HRMS of compound **4** S7
7. ^{1}H NMR of Sensor **1** S8
8. ^{13}C NMR of Sensor **1** S9
9. HRMS of Sensor **1** S10
10. ^{1}H NMR of Sensor **2** S11
11. ^{13}C NMR of Sensor **2** S12
12. HRMS of Sensor **2** S13

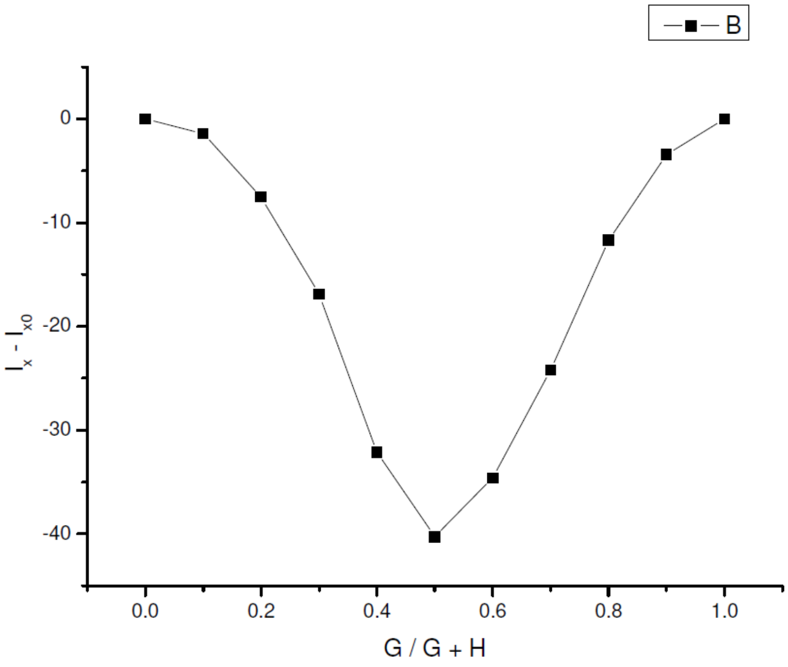

Figure S1: Job plot's of Sensor **1** (at 413 nm) with isophthalate anion.

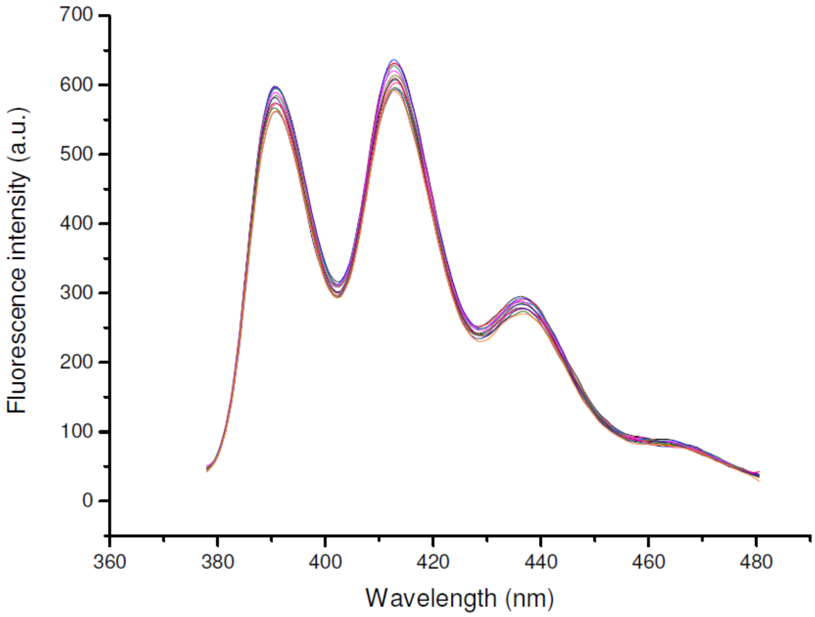

Figure S2: Changes in the fluorescence emission spectra of sensor **1** (5.0×10^{-6} M) upon addition of acetate in acetonitrile. Excitation wavelength = 366 nm.

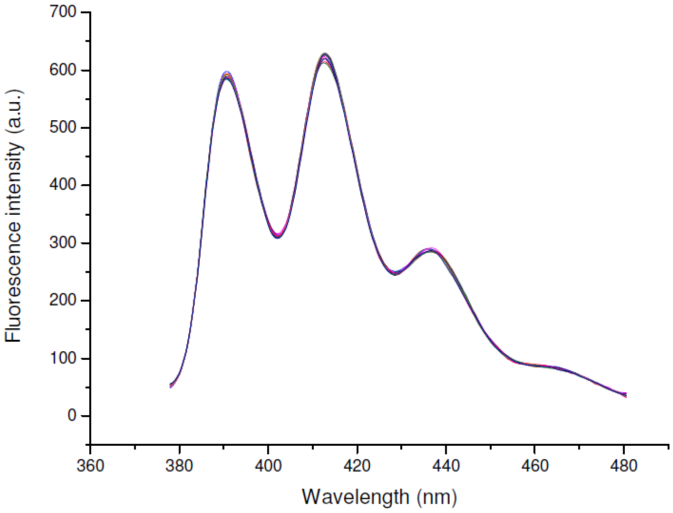

(a)

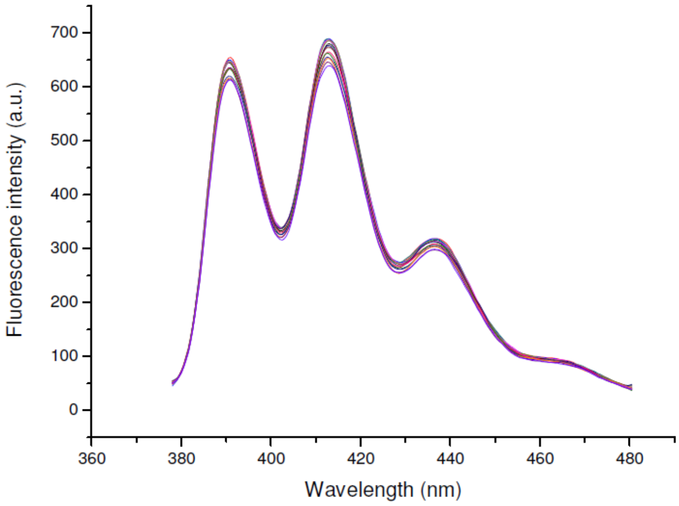

(b)

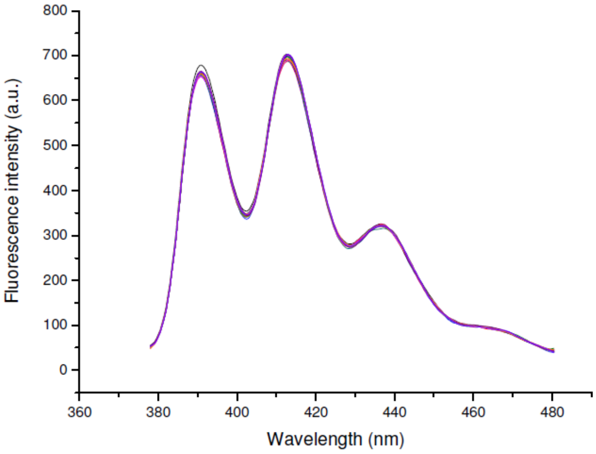

(c)

Figure S3: Fluorescent titration of sensor **1** (5×10^{-6}M) with (a) dihydrogen phosphate; (b) nitrate; (c) bromide (0–20 equiv) in acetonitrile.

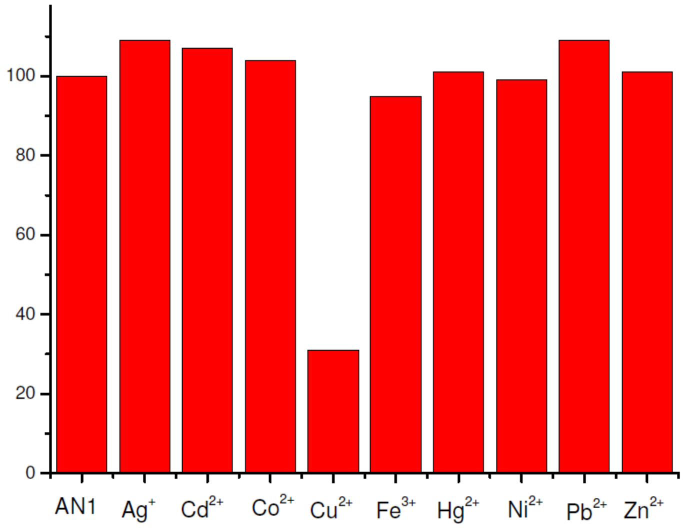

Figure S4: Fluorescence of sensor **AN1** (5×10^{-6}) and **1**–metal mixture (20 equiv of metal ion) in acetonitrile

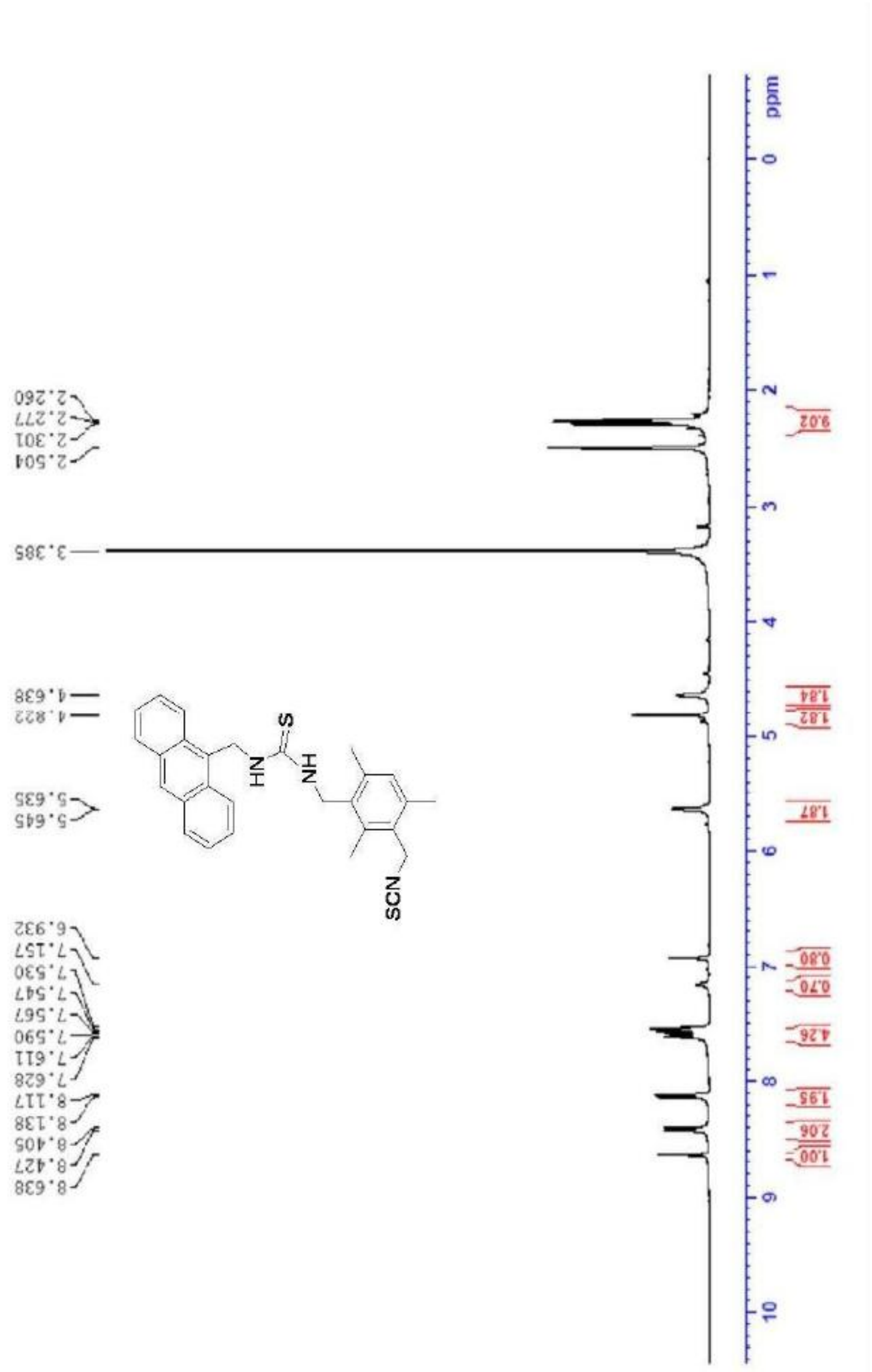
2.260
2.277
2.301
2.504
3.385
4.638
4.822
5.635
5.645
6.932
7.157
7.530
7.547
7.567
7.590
7.611
7.628
8.117
8.138
8.405
8.427
8.638
S
HN
NH
SCN
9.02
1.84
1.82
1.87
0.80
0.70
4.26
1.95
2.06
1.00
ppm

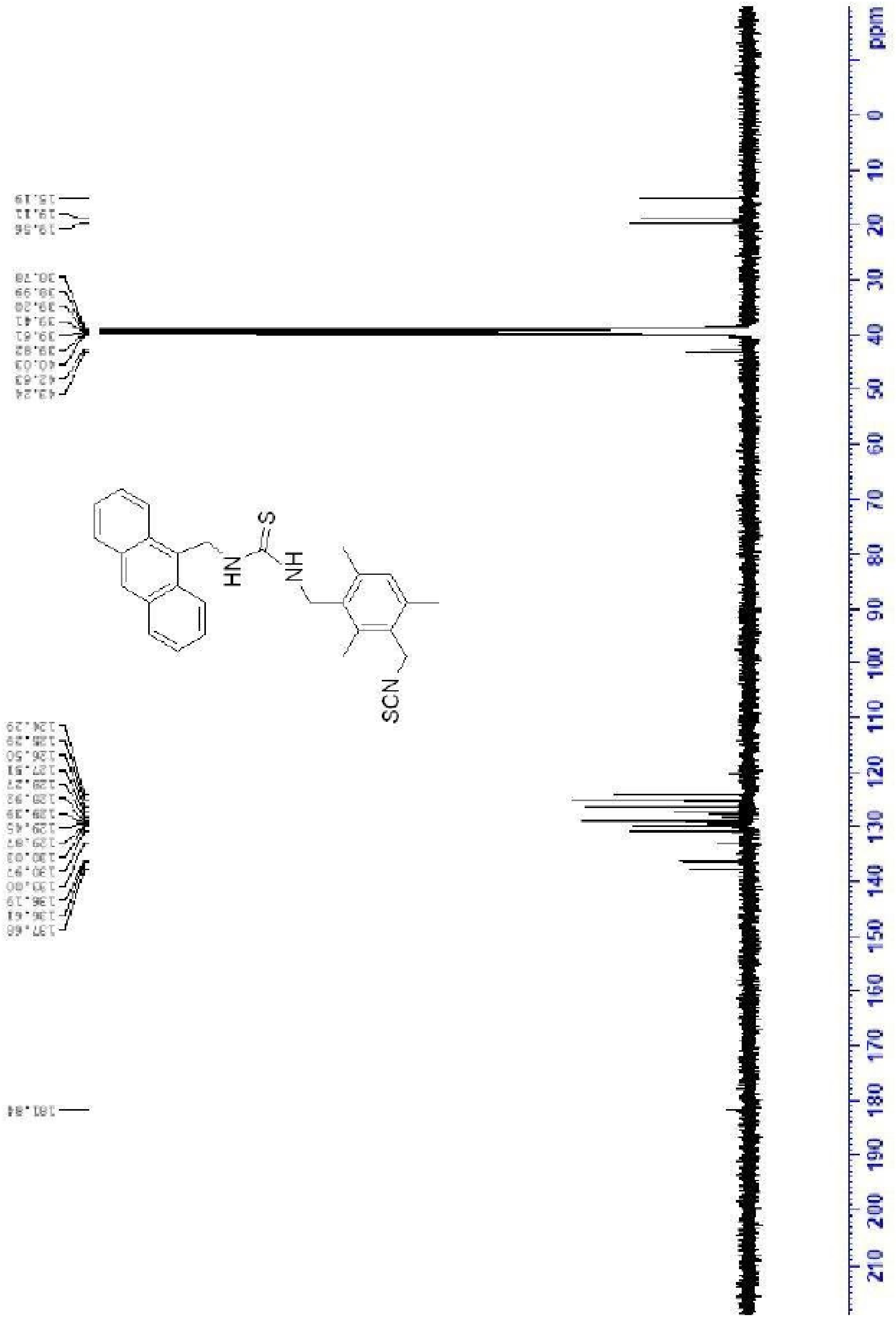

15.19
19.11
19.56
38.78
38.99
39.20
39.41
39.61
39.82
40.03
42.63
43.24
124.29
125.29
126.50
127.51
128.47
128.92
129.39
129.45
129.87
130.03
130.97
135.00
136.19
136.41
137.66
180.84
S
HN
NH
SCN
ppm

HONG KONG BAPTIST UNIVERSITY, DEPARTMENT OF CHEMISTRY (MALDI-TOF)

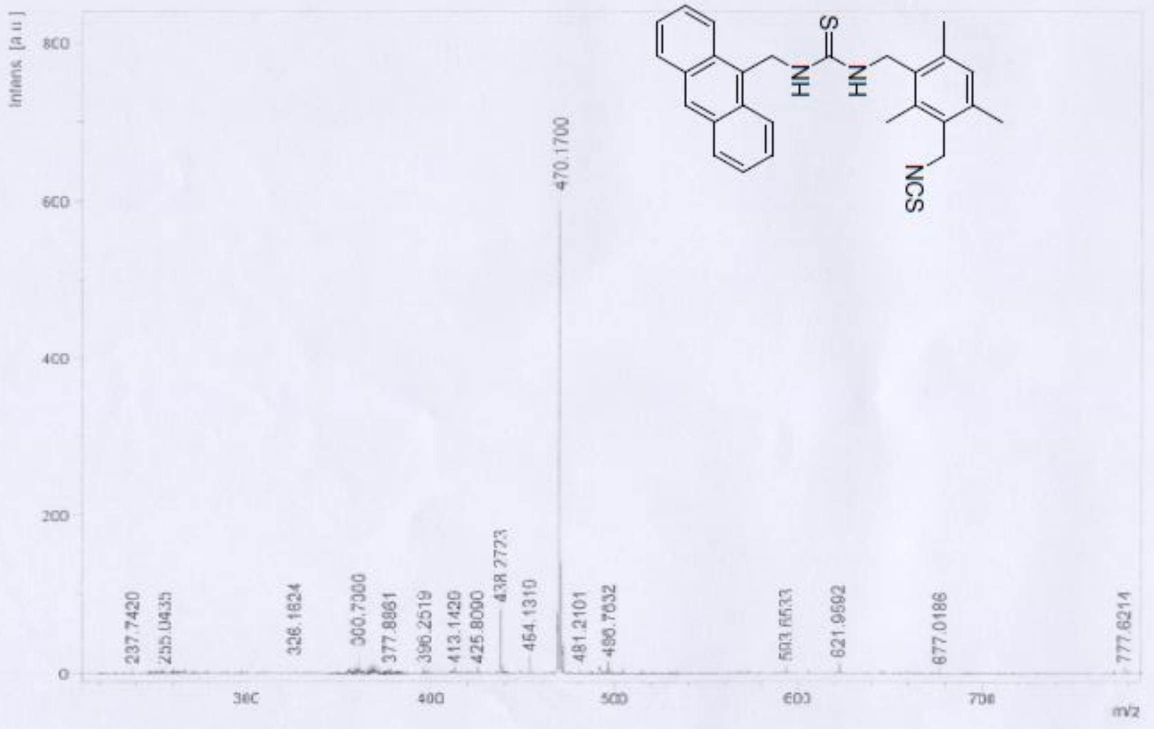

Date of acquisition	[illegible]
Comment 3	Theoretical Value: [illegible]
Instrument type	autoflex
Serial instrument number	[illegible]
PIE delay in [ns]	[illegible] nsec
Voltage polarity	POS
Comment 1	M+H Found: [illegible]
Comment 2	Error [illegible] ppm
Laser repetition rate in Hz	[illegible]
Linear detector voltage	[illegible]
Ion source voltage 1	[illegible]
Ion source voltage 2	[illegible]
Ion source lens voltage	[illegible]
Mass calibration Constant 1	[illegible]
Mass calibration Constant 2	[illegible]
Third calib constant	[illegible]
Number of shots	[illegible]

Acquisition method name [illegible]
Samplename (file name prefix) [illegible]

printed: 01/13/2010 02:44:22 PM

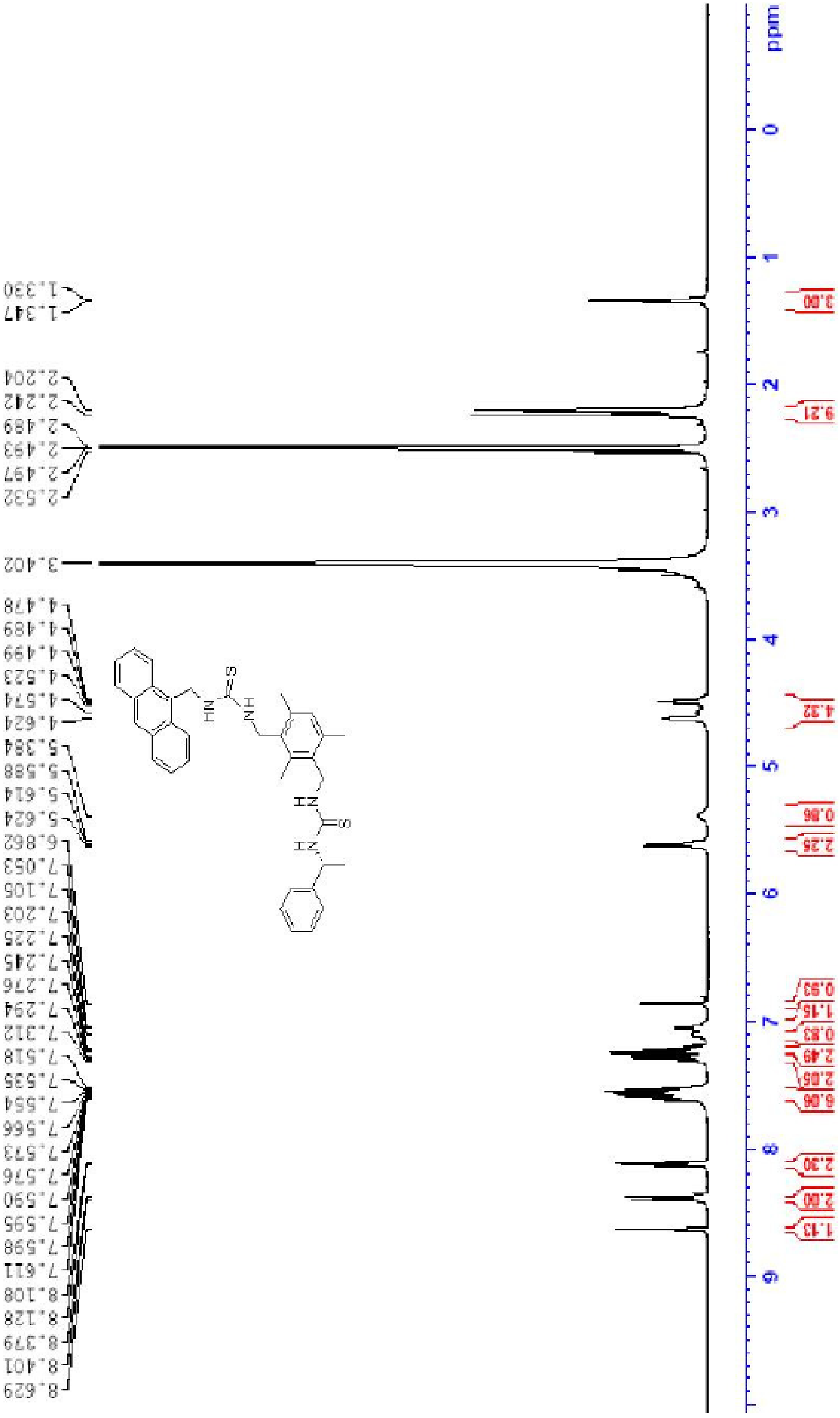
1.330
1.347
2.204
2.242
2.489
2.493
2.497
2.532
3.402
4.478
4.489
4.499
4.523
4.574
4.624
5.384
5.588
5.614
5.624
6.862
7.053
7.106
7.203
7.225
7.245
7.276
7.294
7.312
7.518
7.535
7.554
7.566
7.573
7.576
7.590
7.595
7.598
7.611
8.108
8.128
8.379
8.401
8.629
HN
S
NH
HN
S
NH
ppm
0
1
2
3
4
5
6
7
8
9
3.00
9.21
4.32
0.86
2.35
0.93
1.15
0.83
2.49
2.05
6.06
2.30
2.00
1.13

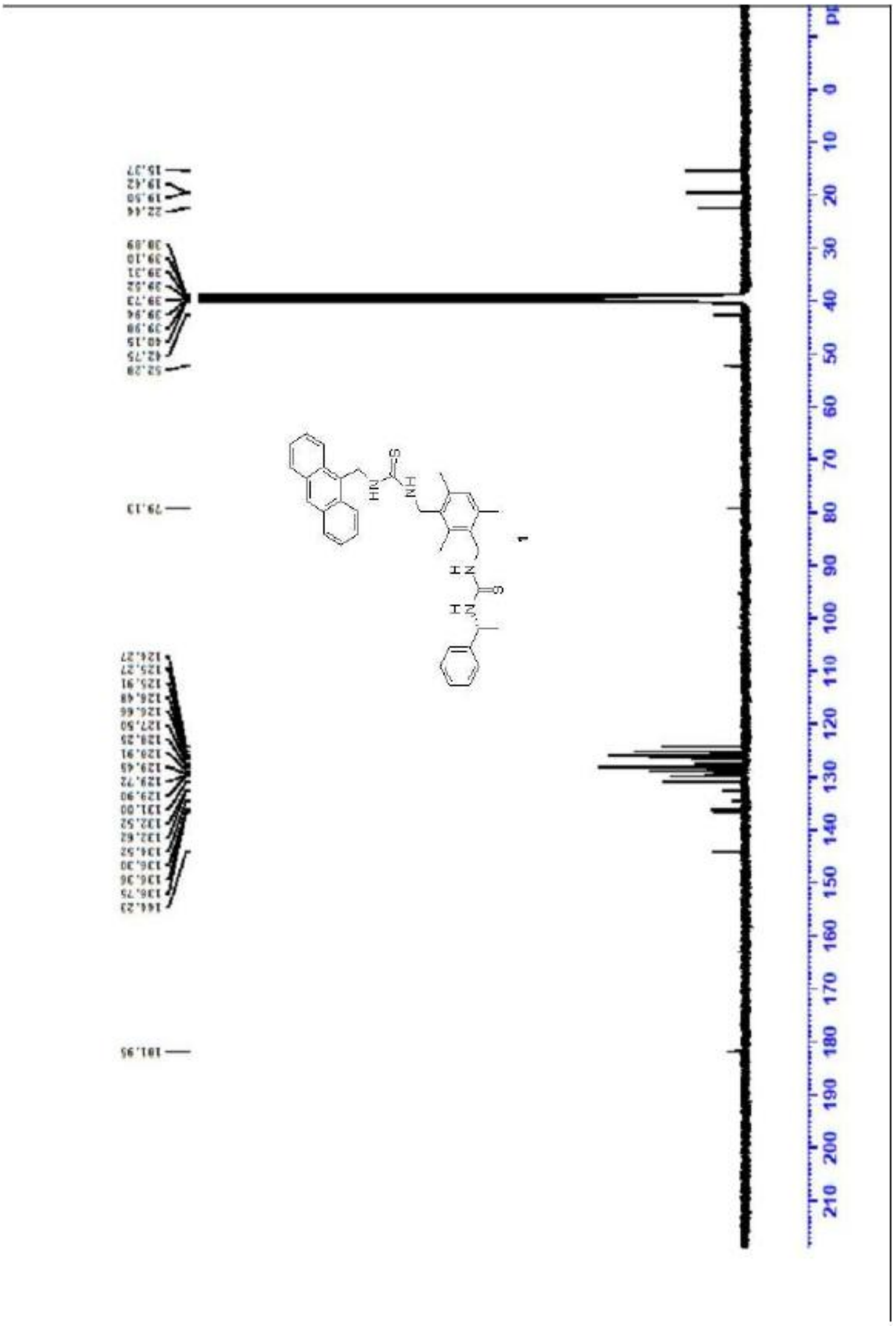

HONG KONG BAPTIST UNIVERSITY, DEPARTMENT OF CHEMISTRY (MALDI-TOF)

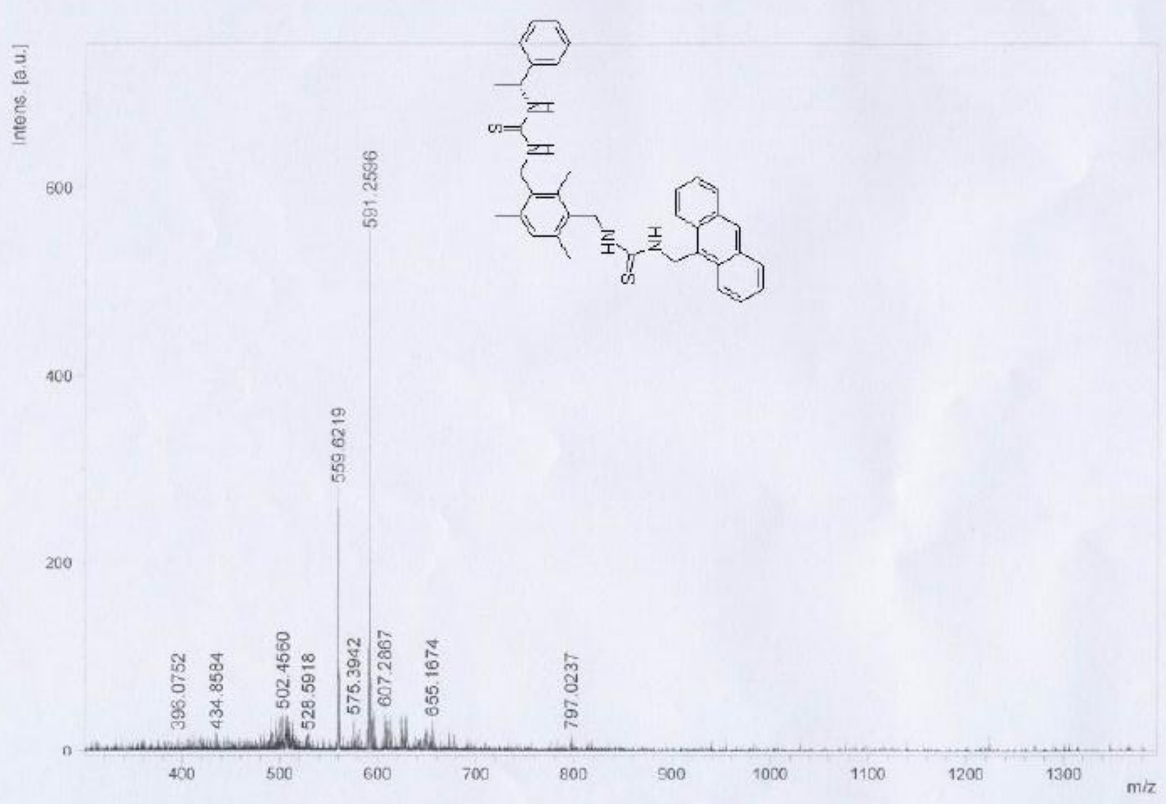

Date of acquisition	2010-01-13 11:42:09
Comment 3	Theoretical Value: 591.2611
Instrument type	autoflex
Serial instrument number	25001.00216
PIE delay in [ns]	140 nsec
Voltage polarity	POS
Comment 1	M+H found: 591.2596
Comment 2	Error: -2.5370ppm
Laser repetition rate in Hz	20 psec
Linear detector voltage	1.711
Ion source voltage 1	19.1
Ion source voltage 2	17.18
Ion source lens voltage	8.3000002
Mass calibration Constant 1	729983.6650695117
Mass calibration Constant 2	138.19261149704l1
Third calib constant	-0.007735147390145288
Number of shots	500

Acquisition method name D:\Methods\flexControlMethods\silvia\RP_Fingerprint 2 12-02-2009.par
Sample name (file name prefix) B-HCCA\0_J18\1

printed: 01/13/2010 11:46:35 AM

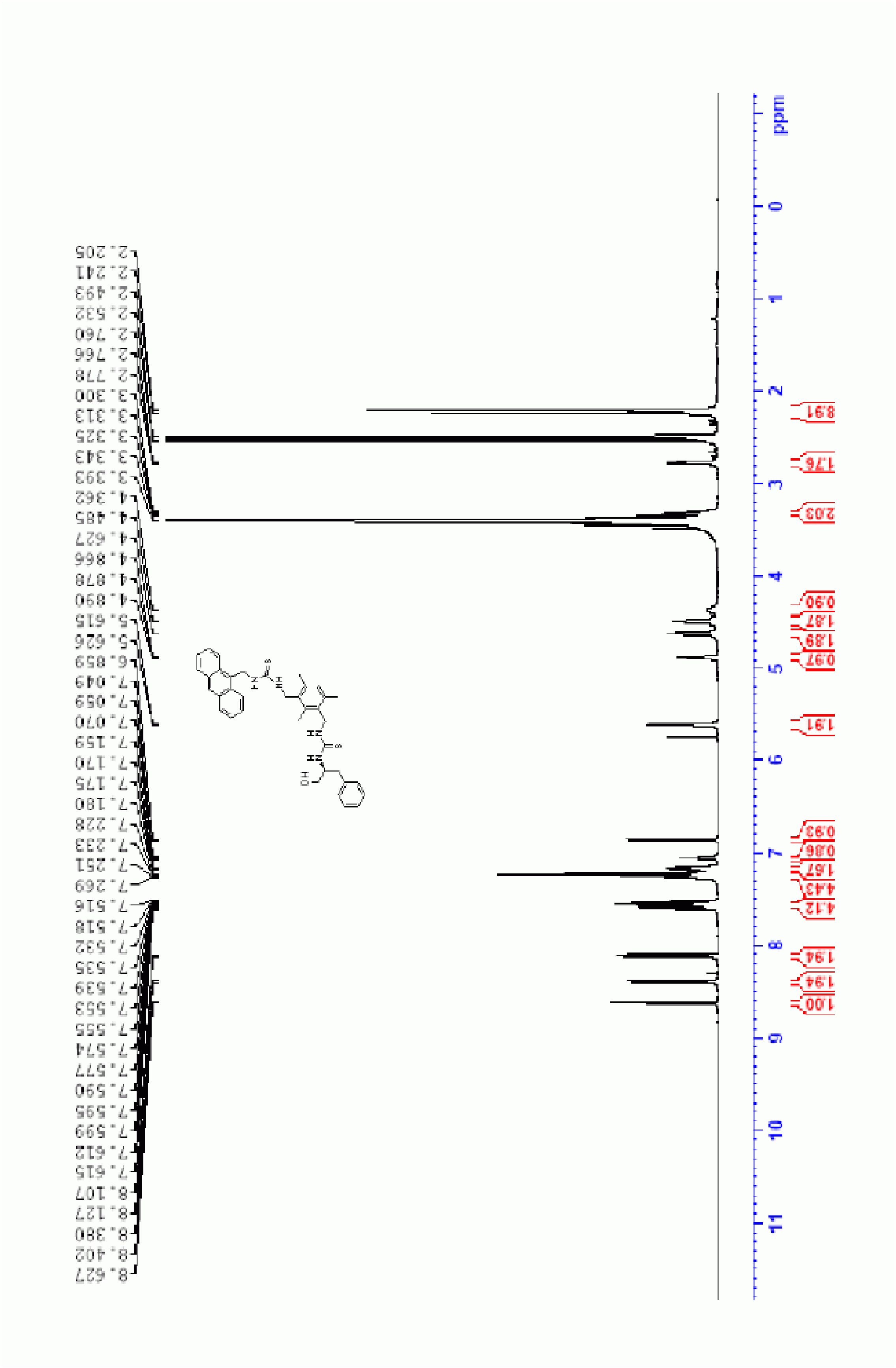
2.205
2.241
2.493
2.532
2.760
2.766
2.778
3.300
3.313
3.325
3.343
3.393
4.362
4.485
4.627
4.866
4.878
4.890
5.615
5.626
6.859
7.049
7.059
7.070
7.159
7.170
7.175
7.180
7.228
7.233
7.251
7.269
7.516
7.518
7.532
7.535
7.539
7.553
7.555
7.574
7.577
7.590
7.595
7.599
7.612
7.615
8.107
8.127
8.380
8.402
8.627
8.91
1.76
2.03
0.90
1.87
1.89
0.97
1.91
0.93
0.86
1.67
4.43
4.12
1.94
1.94
1.00
ppm

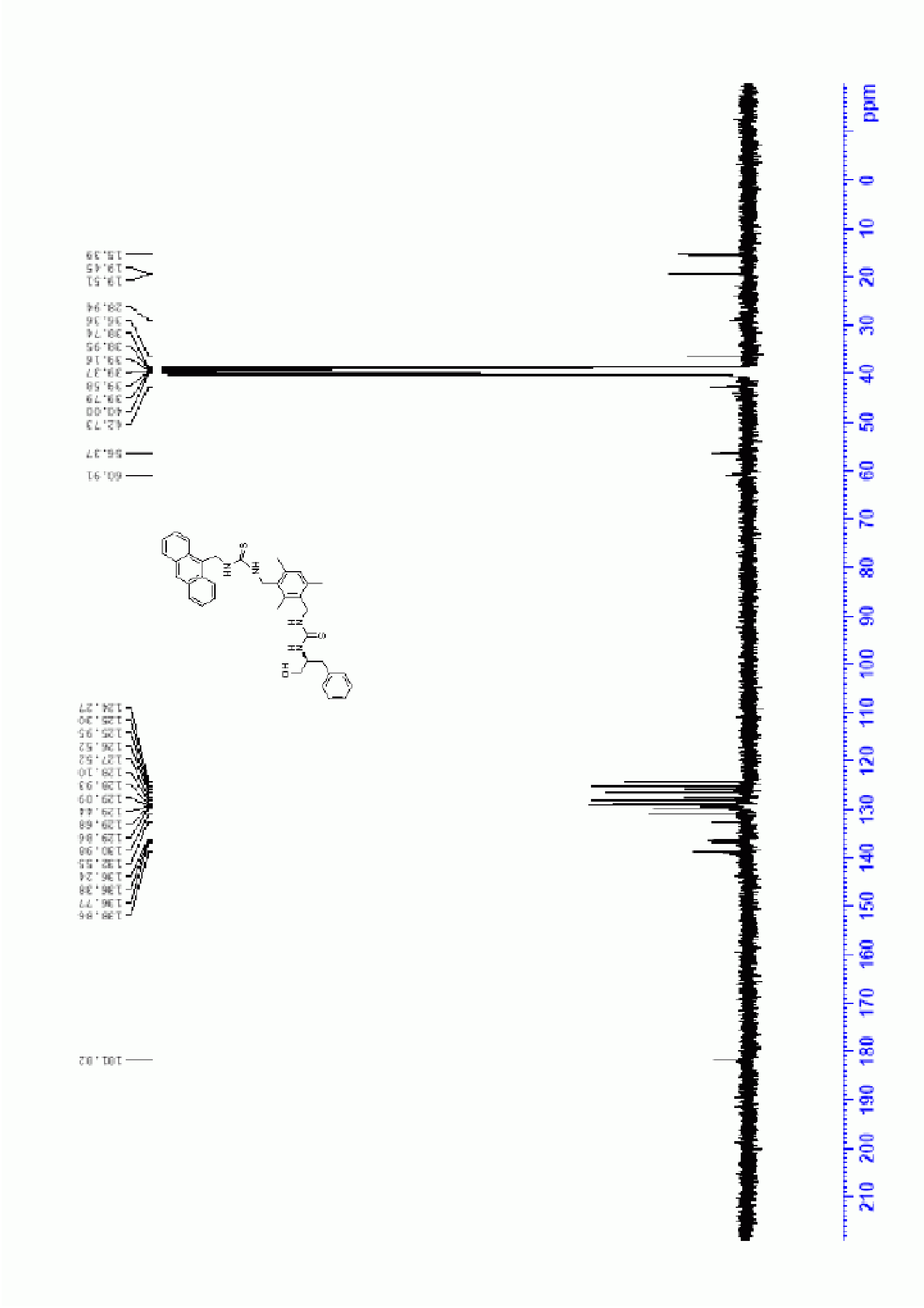

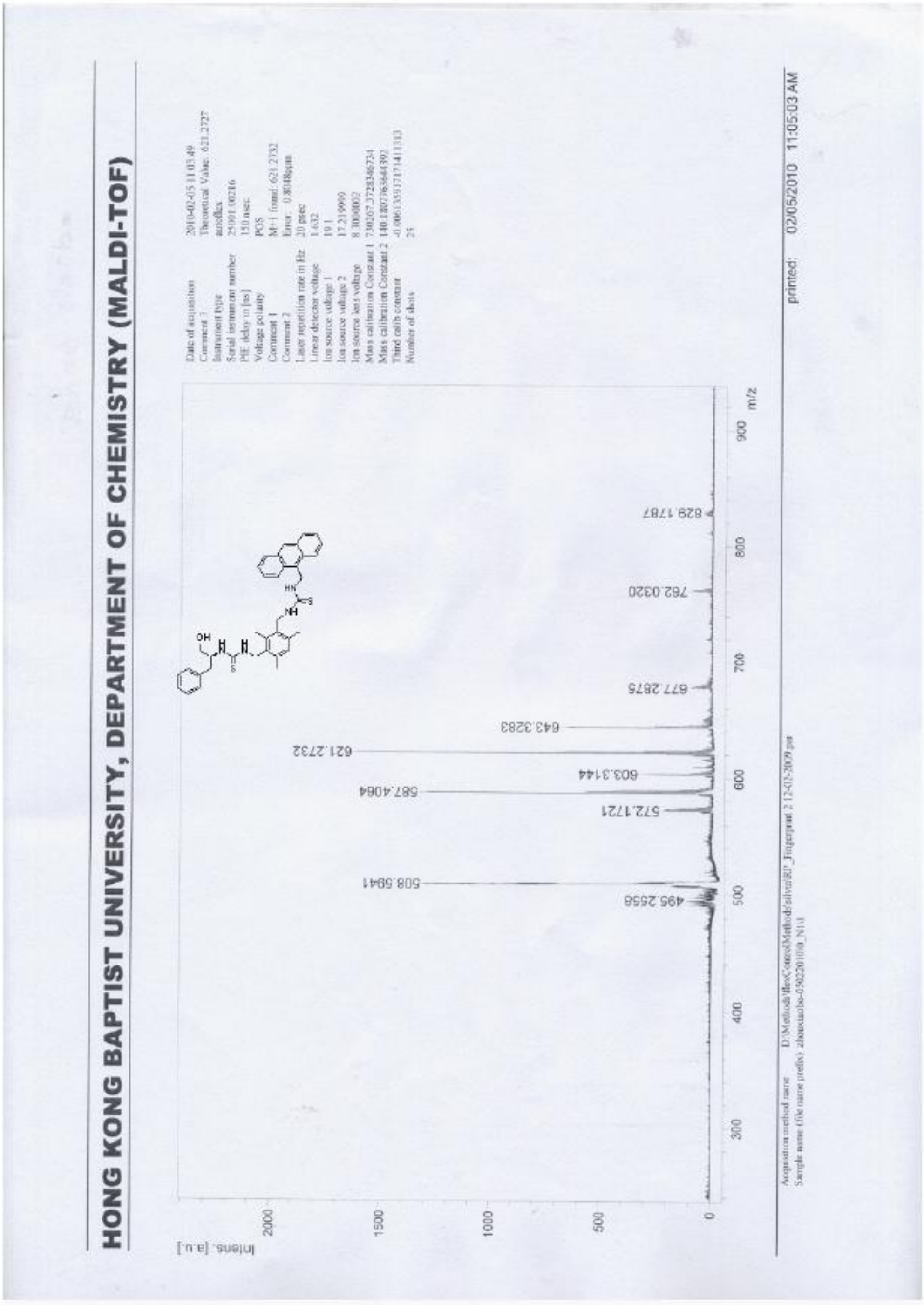
HONG KONG BAPTIST UNIVERSITY, DEPARTMENT OF CHEMISTRY (MALDI-TOF)
Intens. [a.u.]
2000
1500
1000
500
0
300
400
500
600
700
800
900
m/z
495.2558
508.5941
572.1721
587.4084
603.3144
621.2732
643.3283
677.2875
762.0320
829.1787
printed: 02/05/2010 11:05:03 AM
